# Adolescent, but Not Adult, Binge Ethanol Exposure Leads to Persistent Global Reductions of Choline Acetyltransferase Expressing Neurons in Brain

**DOI:** 10.1371/journal.pone.0113421

**Published:** 2014-11-18

**Authors:** Ryan P. Vetreno, Margaret Broadwater, Wen Liu, Linda P. Spear, Fulton T. Crews

**Affiliations:** 1 Bowles Center for Alcohol Studies, School of Medicine, University of North Carolina at Chapel Hill, Chapel Hill, North Carolina, 27599, United States of America; 2 Center for Developmental and Behavioral Neuroscience, Department of Psychology, Binghamton University, Binghamton, New York, 13902, United States of America; Universidade do Estado do Rio de Janeiro, Brazil

## Abstract

During the adolescent transition from childhood to adulthood, notable maturational changes occur in brain neurotransmitter systems. The cholinergic system is composed of several distinct nuclei that exert neuromodulatory control over cognition, arousal, and reward. Binge drinking and alcohol abuse are common during this stage, which might alter the developmental trajectory of this system leading to long-term changes in adult neurobiology. In Experiment 1, adolescent intermittent ethanol (AIE; 5.0 g/kg, i.g., 2-day on/2-day off from postnatal day [P] 25 to P55) treatment led to persistent, global reductions of choline acetyltransferase (ChAT) expression. Administration of the Toll-like receptor 4 agonist lipopolysaccharide to young adult rats (P70) produced a reduction in ChAT+IR that mimicked AIE. To determine if the binge ethanol-induced ChAT decline was unique to the adolescent, Experiment 2 examined ChAT+IR in the basal forebrain following adolescent (P28–P48) and adult (P70–P90) binge ethanol exposure. Twenty-five days later, ChAT expression was reduced in adolescent, but not adult, binge ethanol-exposed animals. In Experiment 3, expression of ChAT and vesicular acetylcholine transporter expression was found to be significantly reduced in the alcoholic basal forebrain relative to moderate drinking controls. Together, these data suggest that adolescent binge ethanol decreases adult ChAT expression, possibly through neuroimmune mechanisms, which might impact adult cognition, arousal, or reward sensitivity.

## Introduction

Adolescence is a neurodevelopmental period in humans and other mammalian species encompassing the transition from childhood to adulthood, and includes increased social interactions, interest in risky behavior (i.e., novelty- and sensation-seeking), and ethanol consumption [1]. Studies of the adolescent brain indicate that many regions continue to undergo significant morphological maturation and refinement of several neurotransmitter systems [2], [3], including the cholinergic system [2]. The cholinergic system is divided into eight nuclei of projection neurons in the basal forebrain and hindbrain (Ch1–Ch8) as well as brain region specific intrinsic interneurons, such as striatal cholinergic interneurons. The basal forebrain consists of the medial septum and vertical limb of the diagonal band (Ch1/2) that project to the hippocampus. The horizontal limb of the diagonal band (Ch3) projects to the olfactory bulbs as well as the piriform and entorhinal cortices. Cholinergic neurons of the nucleus basalis magnocellularis (Ch4) provide major inputs to the entire neocortex and basolateral amygdala, consistent with involvement of the basal forebrain in cognition, executive function, and stress/anxiety states [4]–[7]. Cholinergic cells of the pedunculopontine (Ch5) and the laterodorsal tegmental nucleus (Ch6) project to the thalamus [7], and regulate cortical arousal and behavioral control [8]. The medial habenula (Ch7) projects to the interpeduncular nucleus [9], and plays a role in avoidance learning and behavioral control [10] as well as possibly contributing to habenular regulation of reward pathways [11]. The parabigeminal nucleus (Ch8), which projects to several subcortical visual centers as well as the amygdala [12], is involved in processing of visual information [13]. Thus, continued maturation of the brain cholinergic system during adolescence likely contributes to maturation of multiple brain functions.

Adolescent risk-taking and sensation-novelty seeking coincide with increased experimentation with alcohol and other drugs of abuse [14]. Recent studies find that by 14 years of age, alcohol use has become normative among youth in the United States, with current statistics indicating that approximately 10% of 13–14 year old 8^th^ graders, 25% of 12^th^ graders, and >40% of college students reporting heavy episodic binge drinking (i.e., >5 consecutive drinks per binge drinking episode) [15]. Adolescence and young adulthood are developmental periods with the greatest risk for the onset of alcohol abuse, and adult alcohol use disorders and other drinking problems are associated with a younger age of drinking onset [16]. Developing neurons are generally more sensitive to alcohol and other drugs of abuse, and hence developmental changes occurring in the adolescent brain may imbue adolescence as a vulnerable period of elevated risk for later addictive and other disorders [17]. The developing cholinergic system may be particularly vulnerable. Studies of nicotine exposure suggest that adolescence is a critical period of risk for the developing cholinergic system [18]. Ehlers and colleagues [19] found that adolescent ethanol vapor-exposed rats exhibited reduced ChAT+IR in the basal forebrain (Ch1–4) in adulthood that correlated with increased “disinhibitory” open field behavior as well as altered adult arousal and affective states. These findings support the hypothesis that adolescent binge ethanol exposure persistently alters expression of ChAT+IR cells throughout the cholinergic nuclei of the brain, which might contribute to long-term dysfunction.

We report here that adolescent intermittent ethanol (AIE) decreased ChAT+IR in the majority of cholinergic projection nuclei as well as striatal cholinergic interneurons. Further, we found reductions of basal forebrain ChAT+IR immediately following the conclusion of AIE (postnatal day [P]56) that persisted into later adulthood (P220). Since AIE has been found to increase brain neuroimmune gene expression and signaling, we induced neuroimmune genes using the TLR4 agonist lipopolysaccharide and found a reduction of ChAT+IR that was similar to AIE. Interestingly, a comparison of ethanol exposure in adolescents with comparable exposure in mature adults found that only adolescents undergo a prolonged loss of ChAT+IR following ethanol exposure. Finally, assessment of cholinergic cell markers in the post-mortem human brain revealed diminished protein expression of vesicular acetylcholine transporter and ChAT in the alcoholic basal forebrain, relative to moderate drinking controls. These data suggest that adolescent cholinergic neurons are uniquely sensitive to binge ethanol exposure. Further, a loss of brain cholinergic neurons could contribute to long-term changes in arousal, sleep patterns, cognition, and a variety of other brain functions observed in alcoholism.

## Materials and Methods

### Experiment 1

#### Animals

Young time-mated pregnant female Wistar rats (embryonic day 17; Harlan Sprague-Dawley, Indianapolis, IN) were acclimated to our animal facility prior to birthing at the University of North Carolina at Chapel Hill. On postnatal day (P)1 (24 h after birth), litters were culled to 10 pups. Litters were housed in standard clear plastic tubs with shavings until group housing with same-sex littermates at the time of weaning on P21. All animals were housed in a temperature- (20°C) and humidity-controlled vivarium on a 12 h/12 h light/dark cycle (light onset at 0700 h), and provided *ad libitum* access to food and water. Experimental procedures were approved by the IACUC of the University of North Carolina at Chapel Hill, and conducted in accordance with NIH regulations for the care and use of animals in research.

#### Adolescent Intermittent Ethanol (AIE) Treatment

**Figure 1:**
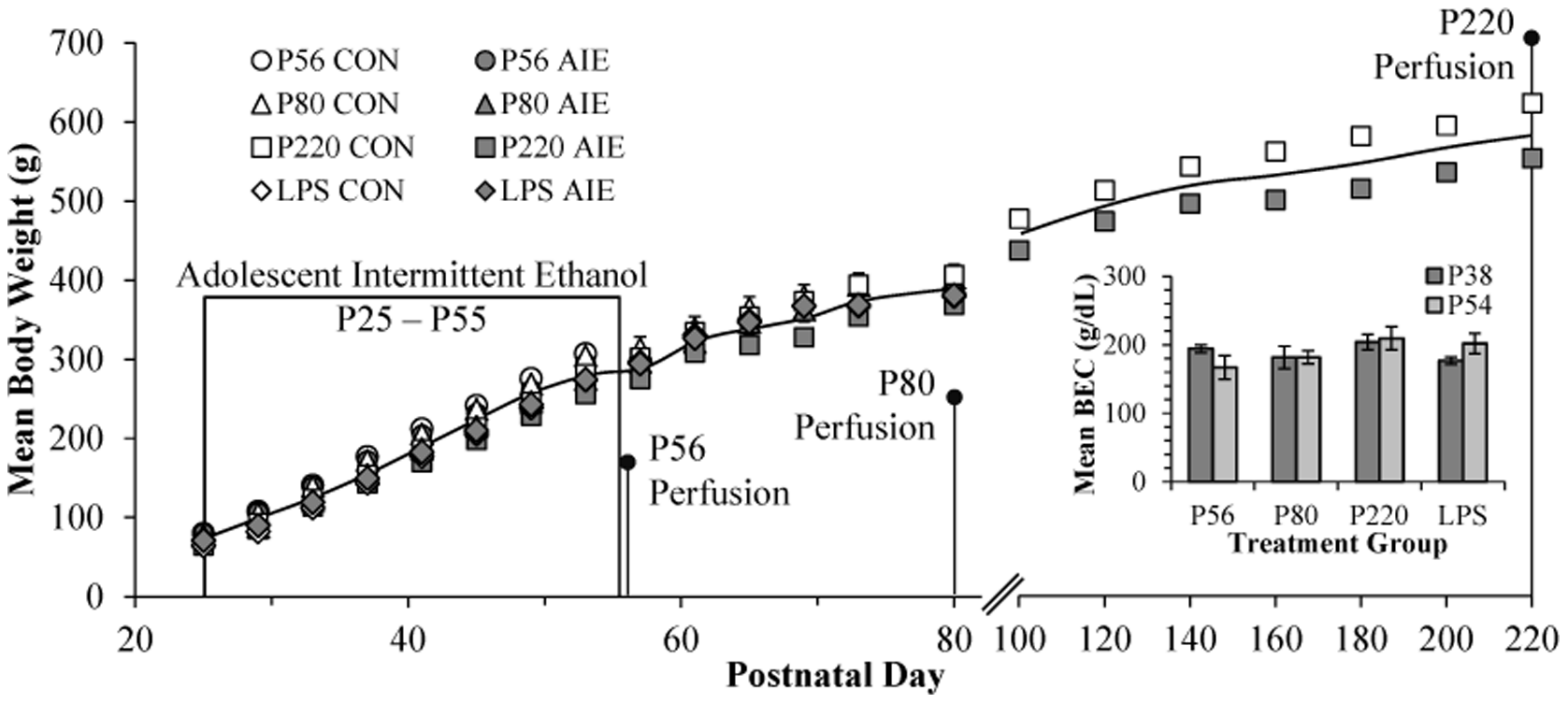
Graphical representation of the adolescent intermittent ethanol (AIE) exposure paradigm. Rats were treated from postnatal day (P) 25 to P55 with either ethanol (5.0 g/kg 20% ethanol v/v, i.g.) or a comparable volume of water on a 2-day on/2-day off administration schedule. Blood ethanol concentration (BEC) was measured one hr after ethanol exposure on P38 and P54. Twenty-four hours, 25 days, and 165 days following the conclusion of AIE, rats were sacrificed for immunohistochemistry.

On P21, male Wistar rats were randomly assigned to either: (i) AIE or (ii) water control (CON). From P25 to P55, AIE animals received intragastric (i.g.) administration of ethanol (5.0 g/kg, 20% ethanol w/v) and CON subjects received comparable volumes of H_2_O on a 2-day on/2-day off schedule. Body weights (g) were measured for the duration of experimentation. Tail blood was collected to assess blood ethanol content (BEC) one h after ethanol administration on P38 and P54, and BECs were calculated using a GM7 Analyzer (Analox; London, UK). On P38 and P54, mean BECs (±SEM) were 189±5 mg/dL and 190±8 mg/dL, respectively, and did not differ across experiments (all *p*'s≥0.2; see Figure 1A insert). For the duration of AIE exposure, subjects evidenced dramatic increases in body weight that did not differ as a function of treatment during AIE exposure (all *p*'s>0.05, P25: CON = 73±1 g, AIE = 73±1 g; P55: CON = 304±6 g, AIE = 286±6 g; P80: 405±8 g, AIE = 389±7 g). However, by P220, AIE-exposed animals weighed significantly less than CON animals (CON = 622±18 g, AIE = 553±17 g [one-way ANOVA: *F* = 7.9, *p*<0.05 (see Figure 1)]).

#### Lipopolysaccharide (LPS) Administration

On P70, AIE- and CON-exposed animals received a single intraperitoneal (i.p.) injection of 1.0 mg/kg LPS (*E. Coli*, serotype 0111:B4; Sigma-Aldrich, St. Louis, MO). Subjects were monitored for 24 h following LPS administration and sacrificed 10 days later on P80.

### Experiment 2

#### Animals

For the adolescent vs. adult intermittent ethanol experiment, male Sprague-Dawley rats were bred and reared at Binghamton University. On P1, litters were culled to 10 pups. Pups were housed in standard clear plastic tubs with shavings until being pair-housed with a same-sex littermate at the time of weaning on P21. All animals were housed in a temperature- (20°C) and humidity-controlled vivarium on a 12 h/12 h light/dark cycle (light onset at 0700 h), and provided *ad libitum* access to food and water. All experimental procedures were approved by the IACUC of the Binghamton University – State University of New York, and conducted in accordance with NIH regulations for the care and use of animals in research.

#### AIE Treatment

On P21, male Sprague-Dawley rats were randomly assigned to either: (i) binge ethanol exposure (BINGE) or (ii) CON. All animals were treated i.g. with ethanol (4.0 g/kg, 25% ethanol v/v) or comparable volumes of H_2_0 every other day either during adolescence (P28 – P48) or young adulthood (P70–P90). After the last exposure day, animals were left undisturbed except for routine animal care and adult Pavlovian fear conditioning, which was previously published [20]. Tissue samples were collected 25 days later for assessment of immunohistochemical assessment of ChAT+IR (see below).

#### Perfusion, Brain Tissue Preparation, and Immunohistochemistry

At the conclusion of EXPERIMENT 1 and 2, animals were anesthetized with sodium pentobarbital (100 mg/kg, i.p.) and transcardially perfused with 0.1 M phosphate-buffered saline (PBS, pH 7.4), followed by 4.0% paraformaldehyde in PBS. Brains were excised and post-fixed in 4.0% paraformaldehyde for 24 h at 4°C followed by 4 days of fixation in 30% sucrose solution. Coronal sections were cut (40 µm) on a sliding microtome (MICROM HM450; ThermoScientific, Austin, TX), and sections were sequentially collected into well plates and stored at −20°C in a cryoprotectant solution (30% glycol/30% ethylene glycol in PBS) for later immunohistochemistry.

Free-floating sections (every 6^th^ section containing the region of interest) were washed in 0.1 M PBS, incubated in 0.3% H_2_O_2_ to inhibit endogenous peroxidases, and blocked with normal goat serum (MP Biomedicals, Solon, OH). Sections were incubated in goat polyclonal anti-ChAT (catalog #AB144P; Millipore, Temecula, CA) for 24 h at 4°C or goat polyclonal anti-vesicular acetylcholine transporter (VAChT; catalog #ABN100; Millipore). Sections were then washed with PBS, incubated for 1 h in biotinylated secondary antibody (1∶200; Vector Laboratories, Burlingame, CA), and incubated for 1 h in avidin-biotin complex solution (Vectastain ABC Kit; Vector Laboratories). The chromagen, nickel-enhanced diaminobenzidine (Sigma-Aldrich, St. Louis, MO), was used to visualize immunoreactivity. Tissue was mounted onto slides, dehydrated, and coverslipped. Negative control for non-specific binding was conducted on separate sections employing the abovementioned procedures with the exception that the primary antibody was omitted.

#### Microscopic Quantification and Image Analysis

**Figure 2:**
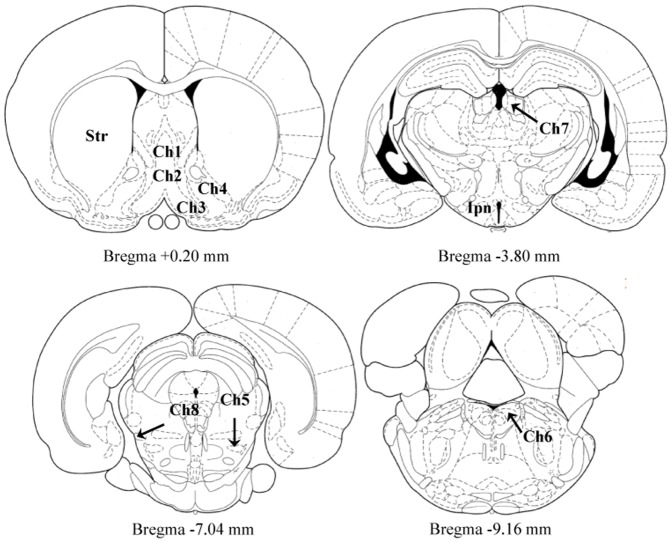
Schematic depicting the location of cholinergic nuclei of the rat brain. Representative micrographs based on the atlas of Paxinos and Watson [21] defining cholinergic brain nuclei assessed for choline acetyltransferase immunoreactivity. Ch1 = medial septum; Ch2 = horizontal limb of the diagonal band; Ch3 = vertical limb of the diagonal band; Ch4 = nucleus basalis magnocellularis; Ch5 = pedunculopontine nuclei; Ch6 = lateral dorsal tegmental nucleus; Ch7 = medial habenula; Ch8 = parabigeminal nuclei; Str = striatum; Ipn = interpeduncular nucleus.

Across studies, BioQuant Nova Advanced Image Analysis (R&M Biometric, Nashville, TN) was used for image capture and analysis. Images were captured with an Olympus UPlan Fl objective (10×/0.30) using an Olympus BX50 microscope and Sony DXC-390 video camera linked to a computer. For each measure, the microscope, camera, and software were background corrected and normalized to preset light levels to ensure fidelity of data acquisition. Assessment of ChAT+IR was performed in Ch1–Ch8, interpeduncular nucleus, and striatum (see Figure 2) according to the atlas of Paxinos and Watson [21]. The total number of ChAT+IR cells was quantified in a 1∶6 series throughout Ch1–Ch6, and striatal tissue samples, and data are expressed as cells per mm^2^. Since ChAT expression was densely distributed throughout Ch7, Ch8, and interpeduncular tissue samples making identification of individual neurons difficult, ChAT+IR pixel density was assessed in a 1∶6 series and rigorously thresholded to normalize pixel intensity [22]. The threshold for pixel density was determined from the control subjects by calculating the average of the darkest and lightest values from each region of interest (i.e., Ch5 [medial habenula], Ch8 [parabigeminal nucleus], and interpeduncular nucleus), and sections were imaged under identical conditions to avoid non-systematic variations [23]. The outlined regions of interest were determined and staining density calculated by dividing the pixel count by the overall area (mm^2^).

### Experiment 3

#### Post-mortem Human Samples

Frozen samples of post-mortem human basal forebrain were obtained from the New South Wales Tissue Resource Centre at the University of Sydney (ethics committee approval number: X11-0107), supported by the National Health and Medical Research Council of Australia-Schizophrenia Research Institute and the National Institute of Alcohol Abuse and Alcoholism (NIH [NIAAA] R24AA012725). Subject information was collected through personal interviews, next-of-kin interviews, and medical records, and is presented in Table 1. Only individuals with alcohol dependence uncomplicated by liver cirrhosis and/or nutritional deficiencies were included. All psychiatric and alcohol use disorder diagnoses were confirmed using the Diagnostic Instrument for Brain Studies that complies with the Diagnostic and Statistical Manual of Mental Disorders [24].

**Table 1:** Case characteristics of human subjects.

Group	Age at Death	Gender	PMI	Clinical Cause of Death
Control	24	Male	43	Undetermined (Consistent with idiopathic cardiac arrhythmia)
Control	43	Male	66	Aspiration pneumonia
Control	44	Male	50	Ischemic heart disease
Control	46	Male	29	Acute myocardial infarction
Control	50	Male	30	Coronary artery disease
Control	50	Male	40	Haemopericardium
Control	53	Male	16	Dilated cardiomyopathy
Control	60	Male	28	Ischemic heart disease
Control	62	Male	46	Ischemic heart disease
Control	48	Male	24	Ischemic heart disease
Alcoholic	49	Male	16	Coronary artery thrombosis
Alcoholic	45	Male	7	Drowning
Alcoholic	49	Male	44	Ischemic heart disease
Alcoholic	61	Male	59	Myocarditis
Alcoholic	51	Male	27	Gastrointestinal hemorrhage
Alcoholic	61	Male	23	Atherosclerotic cardiovascular disease
Alcoholic	42	Male	41	Combined bromoxynil and alcohol toxicity
Alcoholic	50	Male	17	Ischemic heart disease
Alcoholic	44	Male	15	Ischemic heart disease
Alcoholic	43	Male	43	Carbon monoxide intoxication/Alcohol intoxication

PMI: post-mortem interval.

#### Western Blot Analysis

Approximately 75 mg of frozen basal forebrain tissue was homogenized in 0.8 ml RIPA lysis buffer (Sigma-Aldrich) containing protease inhibitor (1∶100; Sigma-Aldrich) and centrifuged at 14,000 rpm at 4°C for 20 min. After centrifugation, protein content in the supernatant was assessed using the Pierce BCA Protein Assay Kit (ThermoScientific, Rockford, IL, Cat # 23227), and 20 µg of protein from each denatured sample was loaded into precast polyacrylamide mini-gel (4–15%; Bio-Rad, Hercules, CA), and blotted onto immunoblot PVDF membranes (Bio-Rad). Immunoblot membranes were blocked in Odyssey blocking buffer (LiCOR Biosciences, Lincoln, NE), and incubated in a primary antibody solution containing either goat anti-ChAT (1∶2000; Millipore) and rabbit anti-tubulin (1∶1000; Cat # 2128; Cell Signaling Technologies, Danvers, MA), mouse anti-VAChT (1∶200; Cat # ab68984; Abcam, Cambridge, MA) and rabbit anti-tubulin (1∶1000), or goat anti-acetylcholinesterase (AChE; 1∶200; Cat # ab31276; Abcam, Cambridge, MA) and rabbit anti-tubulin (1∶1000) for 24 h at 4°C. After washing, the immunoblot membranes were incubated in appropriate fluorescent secondary antibodies (Rockland Immunochemicals, Gilbertsville, PA), and bands were scanned with an Odyssey Infrared Imager (LiCOR Biosciences). Band intensity was quantified using Odyssey Imaging software and normalized to tubulin. All experiments were run in triplicate.

#### Enzyme-linked Immunoabsorbent Assay (ELISA) Analysis

Frozen basal forebrain tissue samples were homogenized in cold lysis buffer (20 mM Tris, 0.25 M sucrose, 2 mM EDTA, 10 mM EGTA, 1% Triton X-100) containing one tablet of Complete Mini Protease Inhibitor Cocktail (Roche Diagnostics, Indianapolis, IN). Homogenates were centrifuged at 5,000× g for five min, supernatant was collected, and protein levels determined using the Pierce BCA Protein Assay Kit (ThermoScientific). The levels of ChAT in the basal forebrain were measured with a human ELISA kit for ChAT from Mybiosource (MBS2020762; San Diego, CA) according to the manufacturer's instruction.

#### Statistical Analysis

Statistical analysis was performed using SPSS (Chicago, IL). Analysis of variance (ANOVA) was used to assess BECs, body weights, immunohistochemistry, Western blot, and ELISA data. To ensure normal distribution of data sets, the Shapiro-Wilk Test of Normality was performed, and data sets identified as non-parametric were analyzed with the independent samples Mann-Whitney U Test. Post-hoc analyses were performed using Tukey's HSD when necessary. Outlier tests were run in each experiment, and any subject with a score exceeding 2 standard deviations greater or less than the group mean was removed from further analysis. No more than one animal was removed from any group. All values are reported as mean ± SEM, and significance was defined as *p*≤0.05.

## Results

### Adolescent binge ethanol exposure reduces ChAT+IR throughout brain cholinergic nuclei

**Figure 3:**
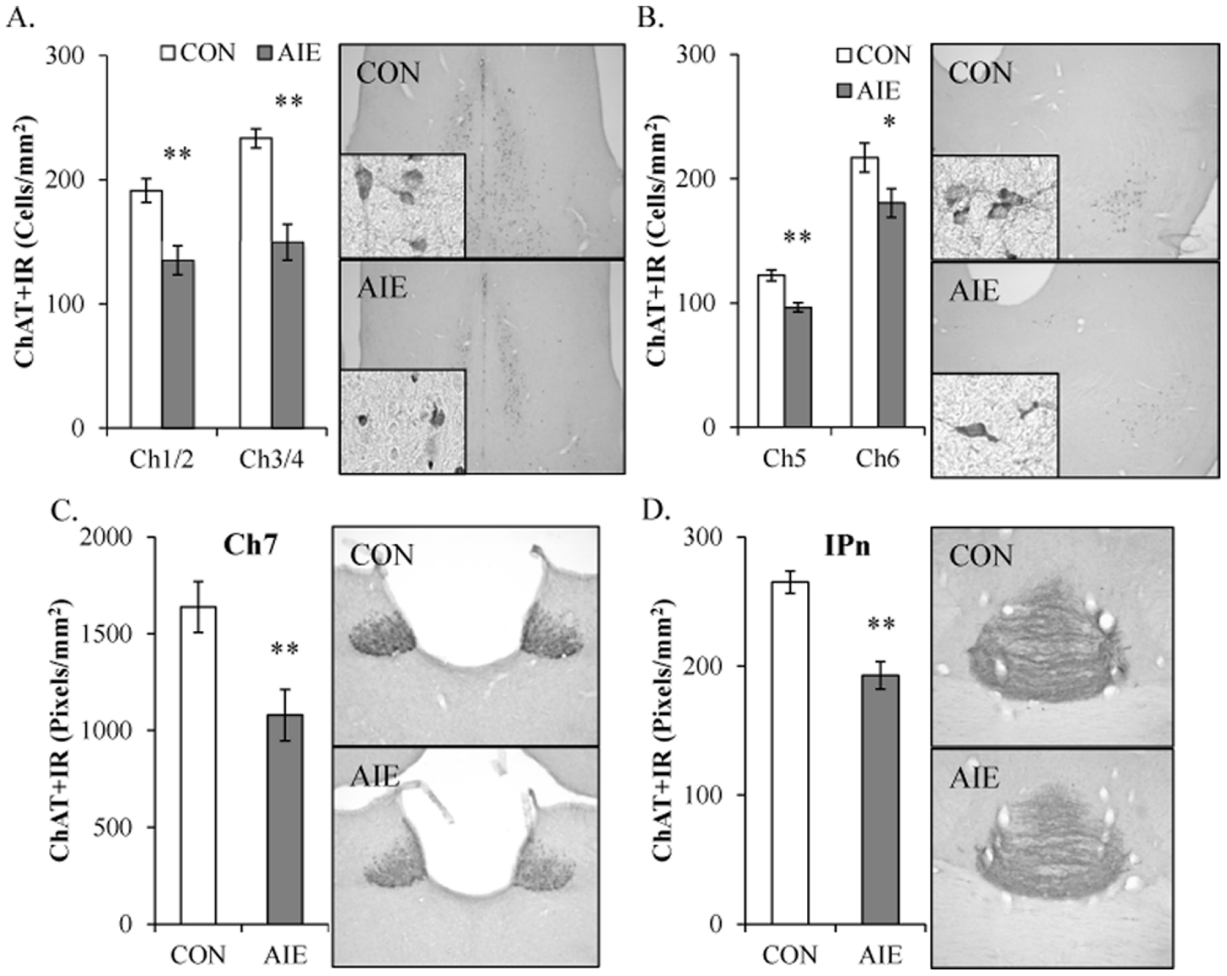
Adolescent intermittent ethanol (AIE) exposure leads to global reductions of choline acetyltransferase (ChAT)-immunopositive cells in the young adult brain. (A) Profile counts revealed that AIE reduced ChAT+IR in the medial septum/vertical limb of the diagonal band (Ch1/Ch2 [29%±4%]) and horizontal limb of the diagonal band/nucleus basalis magnocellularis (Ch3/Ch4 [36%±6%]) of the basal forebrain, relative to controls (CONs). Representative photomicrographs of ChAT+IR cells in Ch1/Ch2 of CON- and AIE-exposed basal forebrain. (B) Profile counts found that AIE reduced ChAT+IR in the pedunculopontine nucleus (Ch5; 21%±3%) and the laterodorsal tegmental nucleus (Ch6; 17%±5%) of the brainstem, relative to CONs. Representative photomicrographs of ChAT+IR cells in Ch5 of the brainstem from CON- and AIE-exposed animals. (C) ChAT pixel density (×1000 pixels/mm^2^) was reduced by 34% (±7%) in the medial habenula (Ch7) of AIE-exposed animals, relative to CONs. Representative photomicrographs of ChAT+IR cells in Ch7 from CON- and AIE-exposed animals. (D) AIE reduced ChAT pixel density (×1000 pixels/mm^2^) by 27% (±4%) in the interpeduncular nucleus (IPn), relative to CONs. Representative photomicrographs of ChAT immunoreactivity cells in the IPn from CON- and AIE-exposed animals. Data are presented as mean ± SEM. * indicates *p*<0.05 and ** indicates <0.01, relative to CON rats.

Choline acetyltransferase, which provides a measure of cholinergic cell populations [25], was assessed in all major cholinergic brain nuclei following adolescent binge ethanol exposure. We model human adolescent drinking behavior using an intermittent schedule consistent with known patterns of heavy weekend binge drinking, but not daily drinking associated with alcoholism in adulthood. Immunohistochemistry was used to assess ChAT+IR in cholinergic projection neurons of Ch1–Ch8 as well as cholinergic interneurons of the striatum and medial habenular (Ch7) terminal fields in the interpeduncular nucleus 25 days after AIE in young adulthood (P80). In CON and AIE basal forebrain samples, assessment of ChAT+IR cells revealed darkly stained cell bodies and processes, with less cell staining in the AIE-exposed animals. Within the basal forebrain, AIE decreased ChAT+IR cell populations by 29% (±4%) and 36% (±6%) in the medial septum/vertical limb of the diagonal band (Ch1/Ch2; one-way ANOVA: *F*_[1,15]_ = 21.1, *p*<0.01) and the horizontal limb of the diagonal band/nucleus basalis magnocellularis (Ch3/Ch4; one-way ANOVA: *F*_[1,15]_ = 20.5, *p*<0.01 [see Figure 3A]), respectively. Thus, AIE reduced ChAT+IR cells in the young adult basal forebrain.

In the brainstem, ChAT+IR was characterized by large and small darkly stained cell bodies and processes. In the pedunculopontine nucleus (Ch5), adolescent binge ethanol reduced ChAT+IR by approximately 21% (±3%), relative to CON animals (one-way ANOVA: *F*_[1,15]_ = 19.4, *p*<0.01). Similarly, in the laterodorsal tegmental nucleus (Ch6), AIE reduced ChAT expression by 17% (±5%) when compared to CON subjects (one-way ANOVA: *F*_[1,15]_ = 4.9, *p*<0.05 [see Figure 3B]). Thus, similar to the basal forebrain, adolescent binge ethanol exposure reduced ChAT+IR in the young adult brainstem cholinergic nuclei.

**Figure 4:**
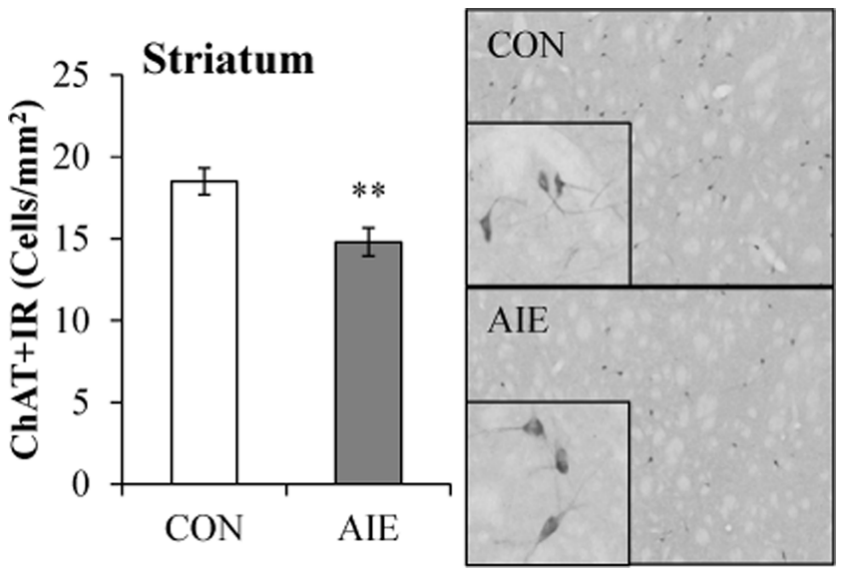
Adolescent intermittent ethanol (AIE) exposure leads to long-term reductions of choline acetyltransferase (ChAT)-immunopositive cells in the young adult striatum. Profile counts of ChAT+IR found a 12% (±3%) reduction of ChAT expression in the striatum of AIE-exposed young adult rats, relative to CONs. Representative photomicrographs of ChAT+IR cells in the striatum from CON- and AIE-exposed animals. Data are presented as mean ± SEM. ** indicates *p*<0.01, relative to CON rats.

Since ChAT expression was reduced in the basal forebrain and brainstem cholinergic nuclei, we also assessed immunoreactivity in the medial habenula (Ch7) and parabigeminal nucleus (Ch8) as well as the interpeduncular nucleus. In the medial habenula (Ch7), ChAT+IR was characterized by well-defined homogenous expression and dense neuropil prompting quantification of ChAT+IR by pixel density. Adolescent binge ethanol exposure reduced ChAT+IR by 34% (±7%) in the medial habenula (Ch7; one-way ANOVA: *F*_[1,15]_ = 10.8, *p*<0.01 [see Figure 3C]), relative to CON animals. The interpeduncular nucleus, a major cholinergic projection site of medial habenula via the fasciculus retroflexus, showed darkly stained ChAT+IR terminal fields in CON- and AIE-exposed animals. Choline acetyltransferase expression was reduced by 27% (±4%) in the AIE animals (one-way ANOVA: *F*_[1,15]_ = 28.1, *p*<0.01 [see Figure 3D]) as compared to CONs. Analysis of ChAT+IR in the parabigeminal nucleus (Ch8) found that AIE did not affect ChAT+IR (*p*>0.6 [data not shown]). In the striatum, ChAT+IR was characterized by darkly stained neurons that were homogenously distributed and ChAT-immunopositive neuron counts were reduced by 20% (±5%) in the AIE animals (one-way ANOVA: *F*_[1,7]_ = 9.5, *p*<0.01 [see Figure 4]), relative to the CONs. Together, these data reveal that binge ethanol exposure during adolescence reduces ChAT+IR in most, but not all, cholinergic brain nuclei in the young adult brain.

**Figure 5:**
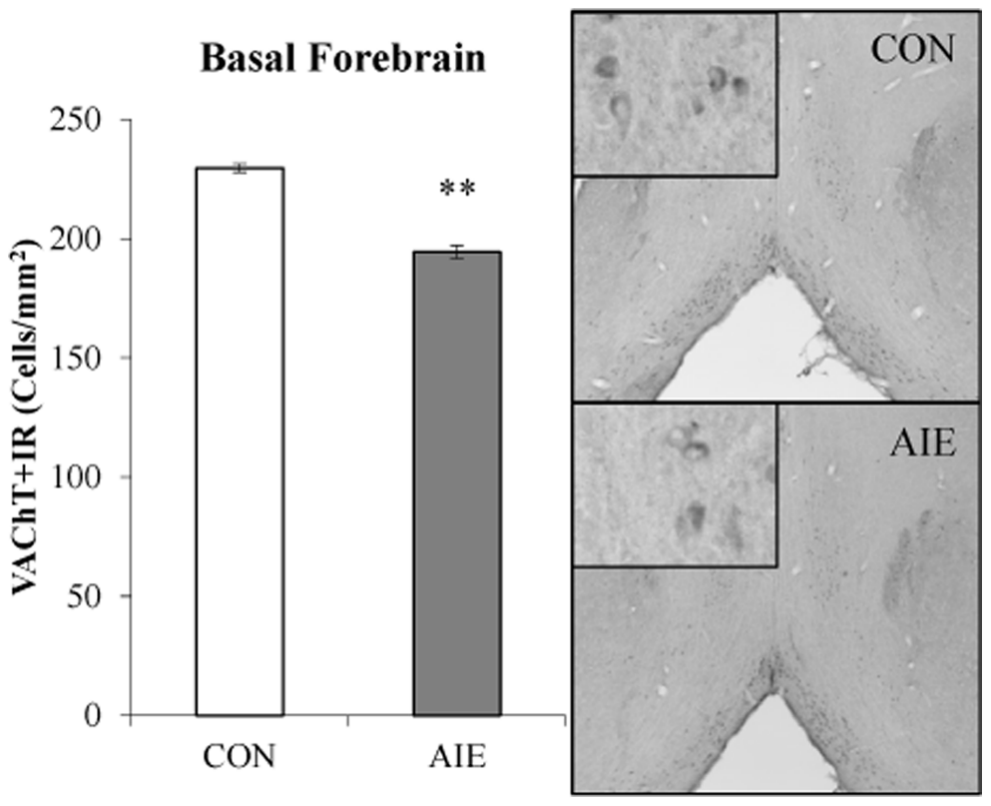
Adolescent intermittent ethanol (AIE) exposure reduces vesicular acetylcholine transporter (VAChT) expression in the young adult basal forebrain. Profile cell counts revealed a 16% (±1%) decrease of VAChT+IR cells in the basal forebrain, relative to controls (CON). Data are presented as mean ± SEM. ** indicates *p*<0.01, relative to CON rats.

To further determine the impact of AIE treatment on cholinergic cells in the young adult basal forebrain, expression of vesicular acetylcholine transporter (VAChT) expression was assessed. VAChT transports cytosolic acetylcholine into synaptic vesicles for storage until release and provides an additional marker for cholinergic cells [26]. In CON and AIE basal forebrain samples, assessment of VAChT+IR cells revealed immunoreactive cell bodies, with less cell staining in the AIE-treated animals. The Shapiro-Wilk Test of Normality was significant for this analysis (*p*<0.05) indicating that the data was not normally distributed. In the young adult basal forebrain, AIE treatment decreased VAChT+IR cell populations by 16% (±1%), relative to CONs (Independent Samples Mann-Whitney U Test: U[13] = 0.0, Z = −3.13, *p*<0.01; see Figure 5). Thus, AIE treatment reduced VAChT+IR cells in the young adult basal forebrain.

### Choline acetyltransferase expression is persistently reduced in the basal forebrain following adolescent binge ethanol exposure

**Figure 6:**
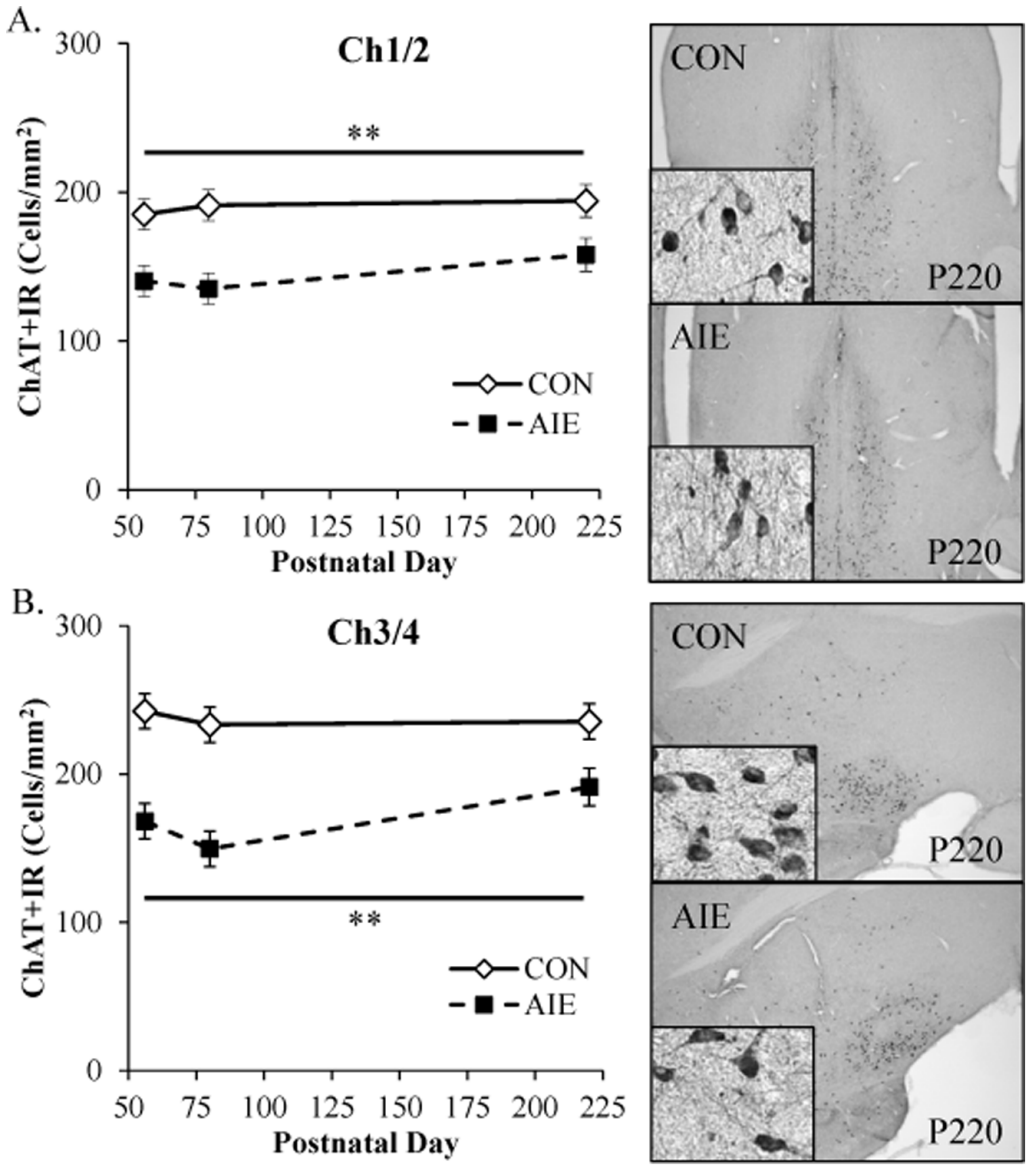
Choline acetyltransferase (ChAT) expression is persistently decreased in the basal forebrain following adolescent intermittent ethanol (AIE) exposure. (A) Profile counts revealed a 24% (±7%) decrease of ChAT+IR cells in the medial septum/vertical limb of the diagonal band (Ch1/Ch2) of adolescent rats (P56) 24 hours following the conclusion of AIE that persisted into adulthood (P220 [19%±3%]), relative to CONs. Representative photomicrographs of ChAT+IR cells in Ch1/Ch2 of CON- and AIE-exposed basal forebrain samples from P220 subjects. (B) Quantification of ChAT immunoreactivity in the horizontal limb of the diagonal band/nucleus basalis magnocellularis (Ch3/Ch4) revealed that AIE reduced ChAT expression by 31% (±4%) at P56 that persisted to P220 (19%±3%), relative to CONs. Representative photomicrographs of ChAT+IR cells in Ch3/Ch4 of CON- and AIE-exposed basal forebrain samples from P220 subjects. Data are presented as mean ± SEM. * indicates *p*<0.05 and ** indicates *p*<0.01, relative to CON rats.

Our finding that adolescent binge ethanol exposure decreased ChAT+IR throughout the young adult brain (P80) prompted us to assess the persistence of the ChAT reductions in the basal forebrain following AIE treatment. Tissue samples were collected from the basal forebrain, and ChAT+IR was assessed in animals sacrificed on P56 (24 h post-AIE treatment), P80 (25 days post-AIE treatment), and P220 (165 days post-AIE treatment). Evaluation of ChAT+IR neurons revealed well defined heterogeneously distributed large and small darkly stained cell bodies and processes in CON and AIE basal forebrain samples (see Figure 6). A 2×3 ANOVA (Treatment [CON vs AIE]×Age [P56 vs P80 vs P220]) found that relative to CON subjects, AIE treatment significantly reduced ChAT+IR in the medial septum/vertical limb of the diagonal band (Ch1/Ch2; main effect of Treatment: *F*_[1,41]_ = 25.2, *p*<0.01; see Figure 6A). Similarly, AIE treatment significantly reduced ChAT expression in the horizontal limb of the diagonal band/nucleus basalis magnocellularis (Ch3/Ch4), relative to CONs (main effect of Treatment: *F*_[1,41]_ = 47.4, *p*<0.01; see Figure 6B). There were no main effects of Age or interactions of Treatment×Age in either analysis. Taken together, these data reveal that adolescent binge ethanol exposure reduces ChAT expression (P56) that persists for long periods (P220), perhaps for the duration of the organism's life.

### Lipopolysaccharide exposure mimics adolescent binge ethanol-induced reductions of ChAT+IR cell populations in the basal forebrain

**Figure 7:**
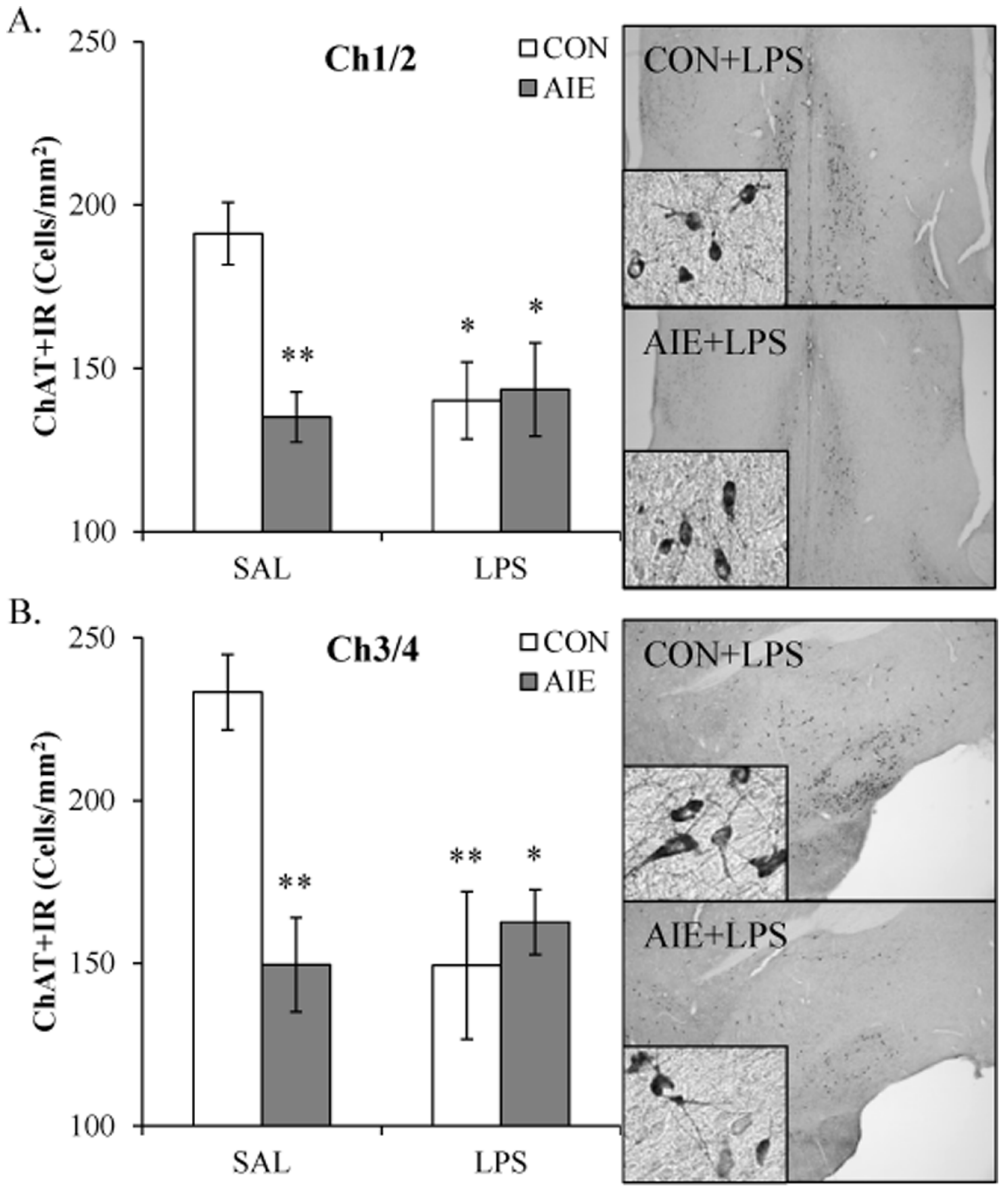
Exposure to lipopolysaccharide (LPS) mimics the choline acetyltransferase (ChAT) reductions associated with adolescent intermittent ethanol (AIE) exposure. (A) Profile counts in the medial septum/vertical limb of the diagonal band (Ch1/Ch2) of young adult rats (P80) revealed a significant reduction of ChAT+IR cells in AIE- (29%±6%), CON+LPS- (27%±6%), and AIE+LPS-exposed (25%±6%) rats, relative to CONs. Representative photomicrographs of ChAT+IR cells in Ch1/Ch2 of CON+LPS- and AIE+LPS-exposed rats. (B) Profile counts in the horizontal limb of the diagonal band/nucleus basalis magnocellularis (Ch3/Ch4) of young adult rats (P80) revealed a significant reduction of ChAT+IR cells in AIE- (36%±7%), CON+LPS- (36%±7%), and AIE+LPS-exposed (30%±7%) rats, relative to CONs. Representative photomicrographs of ChAT+IR cells in Ch3/Ch4 of CON+LPS- and AIE+LPS-exposed rats. Although LPS did not potentiate AIE-induced decreases of ChAT expression, it did result in a similar reduction in CON animals. Data are presented as mean ± SEM. * indicates *p*<0.05 and ** indicates *p*<0.01, relative to CON rats.

Previous research from our laboratory found that adolescent binge ethanol exposure persistently upregulates innate immune signaling and Toll-like receptor 4 (TLR4) expression that persists in the young adult brain [22], [27]. Lipopolysaccharide (LPS) is an endotoxin agonist at TLR4 whose activation leads to proinflammatory cytokine and oxidase induction in brain [28]. To determine if neuroimmune activation contributes to the reduction of cholinergic cells, CON- and AIE-exposed animals received a single dose of LPS (1.0 mg/kg, i.p.) on P70, and ChAT+IR was assessed in the basal forebrain on P80. In CONs, immunohistochemistry revealed darkly stained soma and processes throughout the basal forebrain. ChAT-immunopositive cells in the AIE-exposed animals appeared diminished relative to CONs with a similar staining profile observed in the LPS- and AIE+LPS-exposed animals. Expression of ChAT in the medial septum/vertical limb of the diagonal band (Ch1/Ch2) and the horizontal limb of the diagonal band/nucleus basalis magnocellularis (Ch3/Ch4) of the basal forebrain was analyzed using separate 2×2 ANOVAs (Treatment [CON vs AIE]×Drug [LPS vs SAL]). Within the medial septum/vertical limb of the diagonal band (Ch1/Ch2), AIE treatment significantly reduced ChAT+IR, relative to CONs (main effect of Treatment: *F*_[1,28]_ = 5.6, *p*<0.05). Although there was no main effect of Drug (*p*>0.05), there was a significant interaction (*F*_[1,28]_ = 7.2, *p*<0.05). Post-hoc analysis found that ChAT+IR was reduced following AIE (29% [±6%]; *p*<0.01), CON+LPS (27% [±6%]; *p*<0.05), and AIE+LPS (25% [±6%]; *p*<0.05), relative to CONs (see Figure 7A). Analysis of the horizontal limb of the diagonal band/nucleus basalis magnocellularis (Ch3/Ch4) revealed a significant main effect of Treatment (*F*_[1,28]_ = 4.5, *p*<0.05) and Drug (*F*_[1,28]_ = 4.5, *p*<0.05) as well as a significant interaction (*F*_[1,28]_ = 10.2, *p*<0.01). Post-hoc analysis revealed that AIE (36% [±7%]; *p*<0.01), CON+LPS (36% [±7%]; *p*<0.01), and AIE+LPS (30% [±7%]; *p*<0.05) reduced ChAT+IR, relative to CONs (see Figure 7B). Thus, administration of the TLR4 agonist LPS produced a similar decrease in ChAT+IR neurons in CONs, but did not further reduce ChAT+IR cells in AIE-exposed animals.

### Adolescent, but not adult, binge ethanol exposure reduces ChAT+IR neurons in the basal forebrain

**Figure 8:**
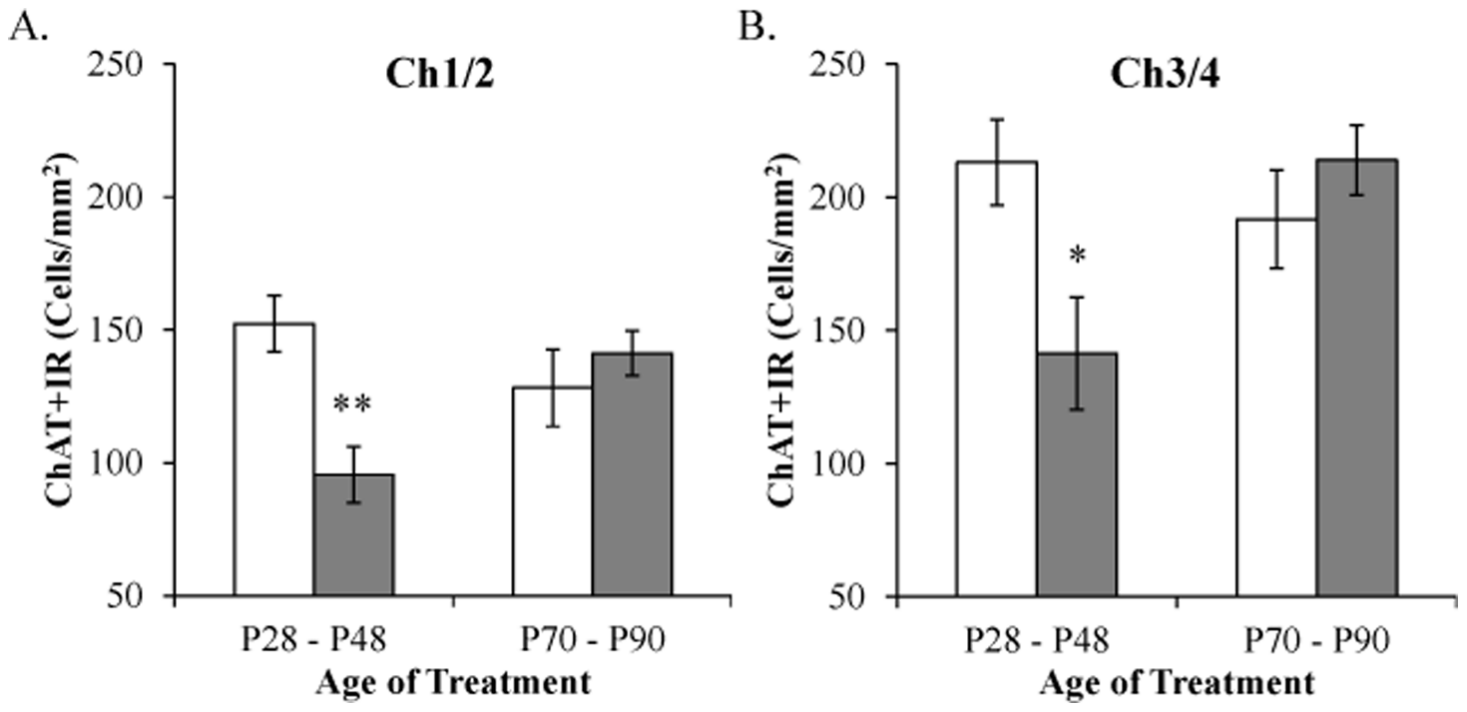
Binge ethanol exposure during adolescence, but not adulthood, decreases choline acetyltransferase (ChAT) expression in the young adult basal forebrain. (A) Immunohistochemical assessment of ChAT+IR in the medial septum/vertical limb of the diagonal band (Ch1/Ch2) revealed a significant 37% (±6%) reduction in subjects that received binge ethanol during adolescence (P28–P48), but not in adulthood (P70–P90). (B) Profile counts of ChAT+IR in the horizontal limb of the diagonal band/nucleus basalis magnocellularis (Ch3/Ch4) revealed a significant 34% (±10%) reduction in subjects that received binge ethanol during adolescence (P28–P48), but not in adulthood (P70–P90). Data are presented as mean ± SEM. * indicates *p*<0.05, relative to CON rats.

Adolescents respond differently to alcohol than adults [3], being particularly sensitive to binge ethanol-induced frontal cortical brain damage [29] and inhibition of neurogenesis [30] while adult neurogenesis is only transiently reduced following binge ethanol exposure [31]. To determine the potential age-associated vulnerability of ChAT+IR cells in the basal forebrain, adolescent (P28–48) and young adult (P70–90) male Sprague-Dawley rats received binge ethanol exposure (4.0 g/kg, 25% ethanol v/v; every other day). In CON- and BINGE-exposed basal forebrain samples, assessment of ChAT+IR cells revealed large and small darkly stained cell bodies and processes in adolescent and young adult animals. Assessment of ChAT+IR in the medial septum/vertical limb of the diagonal band (Ch1/Ch2) with a 2×2 ANOVA (Treatment [CON vs BINGE]×Age [Adolescent vs Young Adult]) did not reveal any main effects (both *p*'s>0.05), but did find a significant interaction (*F*_[1,24]_ = 9.7, *p*<0.01). Post-hoc analyses found that, relative to adolescent CON subjects, ChAT expression was reduced in adolescent binge-exposed rats (37% [±6%]; *p*<0.01), but not adult subjects in either treatment group (both *p*'s>0.4; see Figure 8A). Analysis of ChAT+IR in the horizontal limb of the diagonal band/nucleus basalis magnocellularis (Ch3/Ch4) did not yield any significant main effects (both *p*'s>0.1), but did reveal a significant interaction (*F*_[1,24]_ = 7.3, *p*<0.05). Post-hoc analyses found that, relative to adolescent CON subjects, ChAT expression was reduced in adolescent BINGE-exposed rats (34% [±10%]; *p*<0.05), but not adult subjects in either group (both *p*'s>0.4; see Figure 8B). Our observation of diminished ChAT expression across rat stains (Wistar and Sprague-Dawley) using different exposure parameters indicates that this effect is a general consequence of adolescent binge ethanol exposure. Thus, these data reveal that cholinergic neurons of the adolescent, but not adult, basal forebrain are uniquely vulnerable to the neurotoxic effects of binge ethanol exposure.

### Cholinergic cell marker expression is reduced in the post-mortem human alcoholic basal forebrain

**Figure 9:**
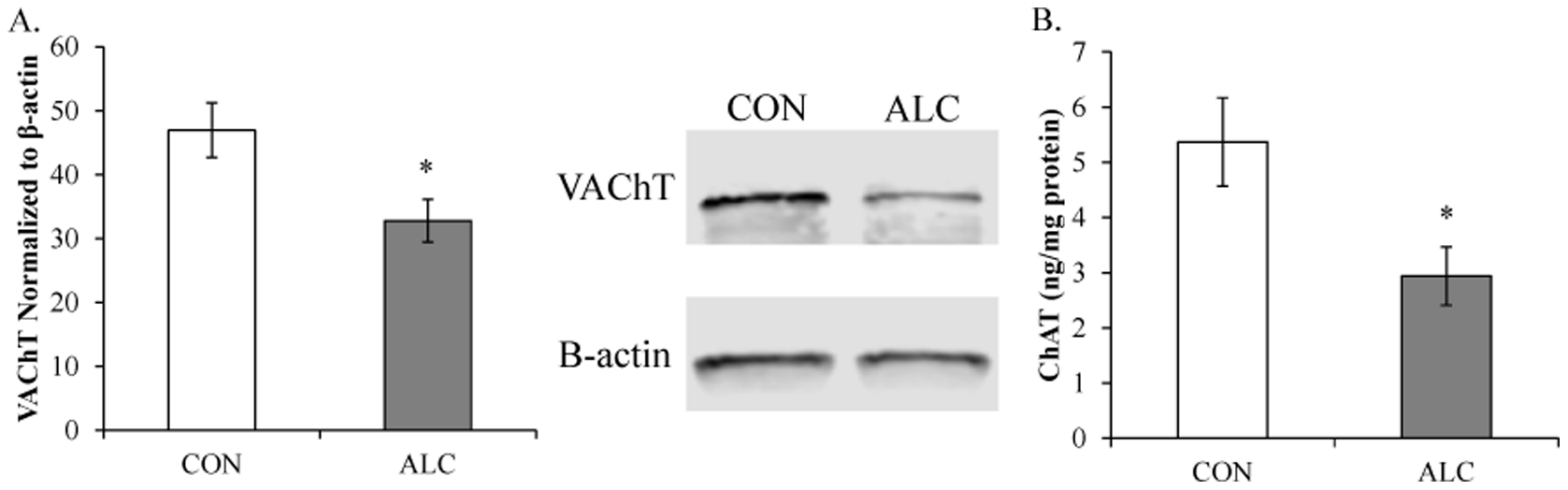
Vesicular acetylcholine transporter (VAChT) and choline acetyltransferase (ChAT) expression is reduced in the human post-mortem alcoholic basal forebrain. (A) Western blot assessment of VAChT protein expression revealed a significant 30% (±7%) reduction in the alcoholic basal forebrain, relative to moderate drinking controls. Representative lanes from Western blot analysis of VAChT. CON is moderate drinking controls and ALC is alcoholic subjects. Western blot analyses were run in triplicate and the mean was reported. (B) ELISA revealed a significant 51% (±11%) reduction of ChAT in the basal forebrain of human alcoholics, relative to moderate drinking controls. Data are presented as mean ± SEM. * indicates *p*<0.05, relative to CONs.

To determine whether cholinergic cell marker expression is altered in the human alcoholic basal forebrain, we first performed Western blot analysis of VAChT, AChE, and ChAT expression in postmortem basal forebrain tissue samples from moderate drinking controls and alcoholic. Analysis of VAChT protein concentrations revealed a significant 30% [±7%] reduction in the postmortem human alcoholic basal forebrain, relative to moderate drinking controls (one-way ANOVA: *F*_[1,16]_ = 6.8, *p*<0.05; see Figure 9A). In contrast, we did not observe a difference in between controls and alcoholics in the amount of AChE expression in the basal forebrain (*p*>0.70). Western blot analysis of ChAT yielded multiple bands at different molecular weights consistent with the presence of multiple slice variants that could not be quantitated reliably prompting us to use ELISA methodologies. ELISA assessment of ChAT protein expression revealed a significant 51% [±11%] reduction in the alcoholic basal forebrain, relative to moderate drinking control subjects (one-way ANOVA: *F*_[1,17]_ = 6.0, *p*<0.05; see Figure 9B). Thus, expression of cholinergic cell marker proteins are reduced in the human alcoholic basal forebrain.

## Discussion

We report here for the first time that protein expression of ChAT and VAChT are reduced in the postmortem human alcoholic basal forebrain in comparison to moderate drinking controls. In our animal model, ChAT immunoreactivity is also reduced in the basal forebrain (Ch1–4) of late adolescent rats (P56) that persists into later adulthood (P220) following exposure to a model of adolescent binge drinking. In addition, we found that ChAT+IR is diminished throughout the young adult (P80) midbrain cholinergic system (Ch5–Ch7) and striatum. In addition to the loss of ChAT+IR in the basal forebrain, expression of VAChT was also reduced in the basal forebrain on P80. Our finding of diminished cholinergic cell protein expression in the human basal forebrain and the persistent loss of cholinergic cell markers in adults rats following adolescent binge ethanol exposure may increase the risk for dysfunction and diseases associated with aging, such as Alzheimer's disease, which also involves a loss of ChAT+IR. Adolescent alcohol abuse is associated with an increased risk for lifetime development of alcoholism [32] and alcohol-associated violence [33]. Thus, these data support the hypothesis that adolescent binge ethanol persistently reduces ChAT expression throughout the brain and these cholinergic changes might contribute to the development of adult psychopathology.

Impaired executive functioning is commonly observed in human alcoholics [34], and the basal forebrain cholinergic system is critically involved in cognitive processes. Indeed, De Rosa and colleagues [35] found that impaired cognitive flexibility in human alcoholics was associated with diminished basal forebrain activation as assessed using fMRI. While they did not directly assess the basal forebrain cholinergic system, our finding that cholinergic cell protein expression is significantly diminished in the human alcoholic basal forebrain might underlie some of the cognitive dysfunction that characterizes alcoholism.

The AIE-associated reduction in ChAT expression throughout Ch1–Ch4 of late adolescent rats (P56) that persisted into adulthood (P220) replicates similar findings in young adult rats and mice exposed to AIE [2], [19]. The cholinergic system of the basal forebrain is an important neuromodulator of memory and attention due to its innervation of the hippocampus and cortex [7], [36]. It undergoes significant refinement during adolescent maturation [2], which increases its vulnerability to the neurotoxic effects of ethanol [37] and these reductions likely contribute to the neurocognitive deficits associated with adolescent binge ethanol exposure. For instance, our laboratory found deficits in reversal learning, which refers to the ability to alter a previously learned behavioral response with a new response when task demands change (i.e., a loss of behavioral flexibility), in young adult rats and mice following AIE exposure [2], [27]. Reversal learning deficits are associated with prefrontal cortex dysfunction, and have also been observed in rats following 192 IgG-saporin lesions of cholinergic cells in the basal forebrain [38]. These deficits might be due to diminished cholinergic innervation of prefrontal cortex structures critical for this form of learning. In addition to involvement of the basal forebrain cholinergic system in cognition, it also plays a significant role in olfactory processes, given the projections from the horizontal limb of the diagonal band (Ch3) to the olfactory bulbs [7]. Olfactory deficits are reported in human alcoholics [39], and loss of cholinergic inputs to this region could also contribute to these deficits [40]. While adolescent binge ethanol exposure has been shown to alter glutamatergic and dopaminergic neuronal functioning [41], previous work from our laboratory found that parvalbumin+IR GABAergic interneurons of the basal forebrain are unaffected in an animal model of AIE [2], suggesting that cholinergic neurons of the basal forebrain are particularly vulnerable to the neurotoxic effects of ethanol. Thus, AIE-induced reductions of ChAT+IR in the basal forebrain may contribute to persistent behavioral flexibility deficits and olfaction in adults and adult alcoholics following adolescent binge drinking.

Brainstem cholinergic neurons, which are distributed throughout Ch5 and Ch6, provide ACh input to the reticular formation, and are involved in the regulation of sleep and wakefulness. Webster and colleagues [42] found that lesions of cholinergic cells within the brainstem disrupted REM sleep. Further, reduction of ChAT expression observed in the pedunculopontine nucleus (Ch5) of a rat model of myocardial infarction was accompanied by diminished REM sleep [43]. Sleep disturbances are associated with a variety of psychopathologies, including alcoholism [44]. In humans, alcohol dependence is associated with sleep disruption both during active drinking as well as abstinence [45]. Our findings of reduced ChAT expression in the pedunculopontine nucleus (Ch5) and laterodorsal tegmental nucleus (Ch6) of young adult rats (P80) after AIE might contribute to lasting sleep disturbances in adults. Indeed, although there are no direct studies on the effects of adolescent ethanol exposure on cholinergic function and sleep, adolescent rats exposed to ethanol vapor from P24 to P60 were found to exhibit diminished slow-wave sleep in adulthood [46].

We also observed reductions of ChAT+IR in the medial habenula (Ch7) and interpeduncular nuclei as well as the striatum. Reductions of ChAT in these regions may increase the risk for development of alcoholism and other drug dependence. The medial habenula-interpeduncular pathway is critically involved in inhibitory control and aversive conditioning [10]. Employing selective ablation techniques to lesion the medial habenula, Kobayashi and colleagues [10] found increased nocturnal hyperactivity as well as increased impulsivity and compulsivity on the 5-choice serial reaction time test. This was accompanied by a 60% reduction of ACh concentration in the interpeduncular nucleus in these mice. Although the present study is the first study to describe AIE-induced reductions of ChAT in the medial habenula and interpeduncular nuclei of young adult rats (P80), nicotine dependence is associated with degeneration of this pathway. Specifically, both continuous and intermittent nicotine exposure increases silver staining, a marker of neurodegeneration, in the medial habenula (Ch7) and the fasciculus retroflexus, a descending fiber pathway that connects the habenula with the interpeduncular nucleus [47], [48]. Interestingly, continuous nicotine exposure at doses that resulted in degeneration of this pathway also increased self-administration of ethanol in rats [49]. Throughout the dorsal and ventral striatum, cholinergic interneurons modulate dopamine release from nerve terminals originating in the substantia nigra and ventral tegmental area, respectively [50], [51]. Both pharmacological depletion of ACh stores in in the striatum with vesamicol, an inhibitor of vesicular ACh transport, and pharmacological blockade of nicotinic ACh receptors, inhibit dopamine release in the striatum [51]. Choline acetyltransferase is essential for the biosynthesis of acetylcholine, and diminished ChAT expression in the striatum might lead to a disruption of the balance between ACh and dopamine. Dysregulation of the neuromodulatory interaction between these two neurotransmitter systems might contribute to compulsive drug taking and addiction. Taken together, disruption of the cholinergic systems of the medial habenula-interpeduncular pathway and striatum might contribute to the development of alcohol dependence through alterations of dopamine-mediated reward responses, diminished inhibitory control, and increased compulsivity. Further research is needed to further elucidate cholinergic interactions with dopaminergic innervation of the striatum as it relates to the development of alcohol dependence.

While the precise mechanism underlying AIE-induced reductions of ChAT expression throughout the brain remain to be fully elucidated, it does appear that the innate immune system might be involved. We previously found persistently upregulated innate immune gene expression in the frontal cortex of young adult rats (P80) exposed to AIE [22], [27]. In the present study, administration of LPS, a prototypical TLR4 agonist, decreased ChAT expression in CON rats to a similar degree to that observed in AIE-exposed animals. Surprisingly, we did not observe a potentiation of ChAT+IR cell loss in the LPS-treated AIE subjects. This is might be due to the vulnerability of a distinct population of basal forebrain cholinergic cells [52], [53] that were diminished during AIE treatment. A consequence of TLR4 activation is induction of oxidative stress due to the production of proinflammatory oxidases [54]. Oxidative stress has been shown to decrease ChAT expression. In basal forebrain cell culture, LPS (10 µg/mL, 2 days) resulted in a pronounced, selective decrease in ChAT-positive neurons, which might be due to nitric oxide (NO) production as microglia produce NO in response to LPS, and the NOS inhibitor NAME blocked the reduction in ChAT expression [55]. In *in vitro* differentiated cholinergic cells models, administration of sodium nitroprusside and aluminum, both of which increase generation of free radicals, reduced ChAT activity. Pyruvate dehydrogenase, an enzyme that converts pyruvate to acetyl-CoA, was also reduced in cultured cholinergic cells following administration of sodium nitroprusside and aluminum [56]. Thus, generation of NO and free radicals might contribute to the reduction of ChAT-immunopositive cells following AIE exposure. Although we find evidence of diminished cholinergic marker expression in the basal forebrain, it is unknown whether ethanol causes cholinergic cell death or down regulation of cholinergic markers. Further research is needed to elucidate this mechanism.

The effects of alcohol on the developing adolescent brain are different from those observed in adulthood. Adolescents are less sensitive to the sedative effects of alcohol [57], but are more vulnerable to alcohol-induced neurotoxicity [37]. In the present study, we found that binge ethanol exposure during adolescence, but not adulthood, led to long-term reductions of ChAT+IR cells in the basal forebrain. Similarly, our laboratory found persistently reduced hippocampal neurogenesis following adolescent, but not adult, binge ethanol exposure [30], [31]. In contrast to the adolescent findings of persistent ChAT loss, chronic ethanol exposure in adulthood transiently reduces cholinergic cell populations in the basal forebrain. In a chronic ethanol exposure model in which rats received 20% ethanol as their sole source of fluid for 12 weeks, expression of acetylcholinesterase (AChE), an enzyme that hydrolyzes ACh into choline and acetyl-CoA, was reduced throughout the basal forebrain immediately following the conclusion of ethanol exposure. In a follow-up study, Arendt and colleagues [58] found progressive reductions of ACh content, release, and synthesis as well as decreased ChAT and AChE activity, and choline reuptake. Interestingly, 4 weeks of abstinence lead to recovery of cholinergic deficits, except in those subjects exposed to 28 weeks of 20% ethanol as their sole source of fluid. Thus, these data support the heightened vulnerability of the developing cholinergic system to the neurotoxic effects of ethanol.

In conclusion, ChAT expression is persistently diminished throughout the adult rat brain following adolescent, but not adult, binge ethanol exposure. Although a mechanism underlying this reduction remains to be fully elucidates, the endotoxin (LPS) data supports the hypothesis that AIE-induced innate immune gene induction contributes to the reduction in ChAT expression. These novel findings suggest that some of the behavioral deficits observed in humans and in animal models of adolescent binge drinking might be the result of a global cholinergic dysfunction. These novel findings reveal that early life insults can have long-lasting effects on the maturing cholinergic system of the CNS and might contribute to some of the behavioral deficits observed in humans and in animal models of adolescent binge drinking.
